# Water-soluble Manganese and Iron Mesotetrakis(carboxyl)porphyrin: DNA Binding, Oxidative Cleavage, and Cytotoxic Activities

**DOI:** 10.3390/molecules22071084

**Published:** 2017-06-29

**Authors:** Lei Shi, Yi-Yu Jiang, Tao Jiang, Wei Yin, Jian-Ping Yang, Man-Li Cao, Yu-Qi Fang, Hai-Yang Liu

**Affiliations:** 1 Department of Chemistry, Guangdong University of Education, Guangzhou 510303, China; jt@gdei.edu.cn (T.J.); yinwei@gdei.edu.cn (W.Y.); yangjianping@gdei.edu.cn (J.-P.Y.); caomanli@gdei.edu.cn (M.-L.C.); 15622197246@163.com (Y.-Q.F.); 2 Engineering Technology Development Center of Advanced Materials & Energy Saving and Emission Reduction in Guangdong Colleges and Universities, Guangzhou 510303, China; 3 Department of Chemistry, South China University of Technology, Guangzhou 510641, China; chemjiangyiyi@163.com

**Keywords:** porphyrin, manganese, iron, DNA, cytotoxicity

## Abstract

Two new water-soluble metal carboxyl porphyrins, manganese (III) *meso*-tetrakis (carboxyl) porphyrin and iron (III) *meso*-tetrakis (carboxyl) porphyrin, were synthesized and characterized. Their interactions with ct-DNA were investigated by UV-Vis titration, fluorescence spectra, viscosity measurement and CD spectra. The results showed they can strongly bind to ct-DNA via outside binding mode. Electrophoresis experiments revealed that both complexes can cleave pBR322 DNA efficiently in the presence of hydrogen peroxide, albeit **2-Mn** exhibited a little higher efficiency. The inhibitor tests suggest the oxidative DNA cleavage by these two complexes may involve hydroxyl radical active intermediates. Notably, **2-Mn** exhibited considerable photocytotoxicity against Hep G2 cell via triggering a significant generation of ROS and causing disruption of MMP after irradiation.

## 1. Introduction

**Figure 1:**
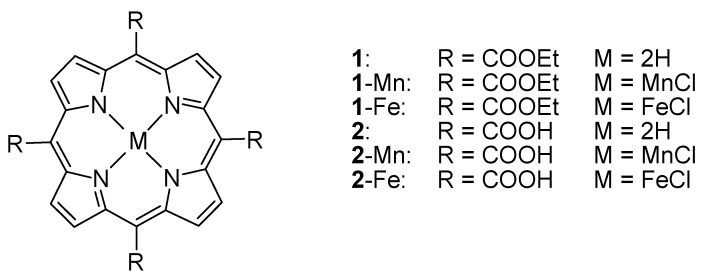
Molecular structures of porphyrin derivatives.

Porphyrins or metalloporphyrins are known as potential chemotherapeutical agents for anticancer therapy, because they can preferentially accumulate in tumor tissues [1,2,3,4,5,6]. Porphyrin derivatives show a range of applications in biology and medicine, such as photodynamic therapy [7,8], anticancer agents [9,10], and tumor imaging [10,11]. Water-soluble porphyrins have been considered as one of the most promising anticancer drugs owing to their water solubility, low dark toxicity, high affinity for tumor sites and strong DNA-binding affinity [3,12,13,14]. Neutral porphyrins conjugated with amino acids have also been considered as potential photosensitizers for PACT [15] while their interactions with DNA have attracted less much attention [16]. On the other hand, water soluble cationic porphyrins have been proved to display good photo and/or chemical nuclease activity with antiviral properties [17]. Manganese (III) and iron (III) cationic porphyrins are very efficient oxidative DNA cleavage agents. What’s more, manganese and iron are also abundant on Earth, cost effective, and environmentally benign [18,19]. So far, lots of metal (Ag, Zn, Co, Ni, Pb, Fe, Mn, Cu, Au, Hg, Cd) cationic porphyrin complexes have been synthesized and tested as potential chemotherapeutical agents [6,20,21,22,23,24], and even showed higher potency than cisplatin [25,26]. Meanwhile, anionic sulfonated porphyrins have been known exhibit highly efficient in internalizing and eliciting severe damage in cancer cells upon photo irradiation [27]. What’s more, they also have the activity against the human immunodeficiency virus (HIV-1) [28], and strong phototoxic effect against Tobacco Bright Yellow-2-cells [29]. Anionic carboxyl porphyrins also show good DNA binding and cleavage activity [30,31,32]. However, less information about biological studies on anionic porphyrins are available [27,28,29,30,31,32,33,34]. Previous studies have confirmed that anionic porphyrins have better membrane permeability than cationic ones [35,36,37], which may explain their higher biological activity. In order to extend the biological application scope of anionic porphyrins, two new water-soluble anionic manganese (III) and iron (III) *meso*-tetrakis (carboxyl) porphyrin were prepared by hydrolysis of their corresponding 5,10,15,20-tetrakis (ethoxycarbonyl) porphyrin metal complexes [38,39] (Figure 1). The DNA binding and nuclease activity of the anionic complexes were examined by various spectral, viscosity and gel electrophoresis measurements. The inhibitory growth activities were evaluated by MTT assays.

## 2. Results and Discussion

### 2.1. Synthesis and Characterization

The synthesized porphyrins were well characterized by UV-Vis spectra (Figures S1 and S2, see Supplementary Materials). The characteristic Soret band for free base porphyrinis is observed at 407 nm, and the Q-bands are observed at 505, 540, 583, 638 nm, while the metal insertion into the core of porphyrin alters the optical spectrum drastically. Manganese porphyrin **1-Mn** exhibits a Soret band at 364 nm, and a Q band at 570 nm, as well as a metal to ligand charge transfer transition (MLCT) band at 474 nm. Various reports have emphasized the formation of μ-oxo iron porphyrin dimer during the metalation of free base porphyrin [19].

**Figure 2:**
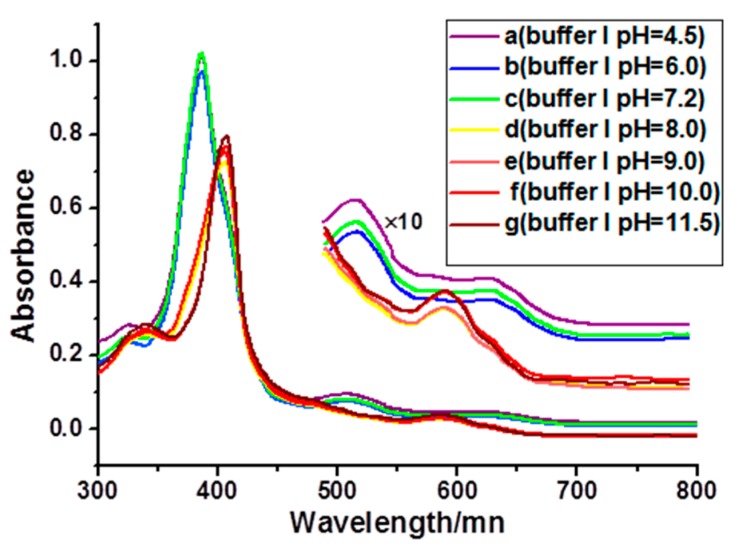
The UV-Vis spectral changes of **2-Fe** in 5 mM Tris-HCl/50 mM NaCl buffer at different pH. Inset: enlarged Q bands.

The UV-Vis spectra of iron porphyrin monomer display distinct characteristics as compared with the dimer. Normally, the Soret band graphs of iron porphyrin monomer and μ-oxo dimer are nearly the same, whereas there are some differences in the Q band. As compared to the monomer, the Q band will disappear, and a new band will appear in the μ-oxo dimer. The μ-oxo dimer-monomer interconversion of iron porphyrin is strongly dependent on the pH value [40]. Figure 2 shows the UV-Vis spectral changes of iron porphyrin stock solution in freshly prepared buffer I solution with different pH values. It can be seen that the Soret band of **2-Fe** in buffer I with pH = 8.0–11.5 (Figure 2d–g) exhibit remarkable red shift as compared to pH = 4.5–7.2 (Figure 2a–c), and the Q bands are also different. These observations suggest **2-Fe** exists in buffer I with pH =8.0–11.5 as μ-oxo dimer, and it may exist as a monomer in buffer I with pH values varying from 4.5–7.2 [40]. In addition, all compounds were also fully characterized by NMR and HR-MS spectroscopy (Figures S3–S11) and the purities of **2**, **2-Mn**, **2-Fe** were determined by HLPC (Figure S12).

### 2.2. DNA Binding Properties

**Figure 3:**
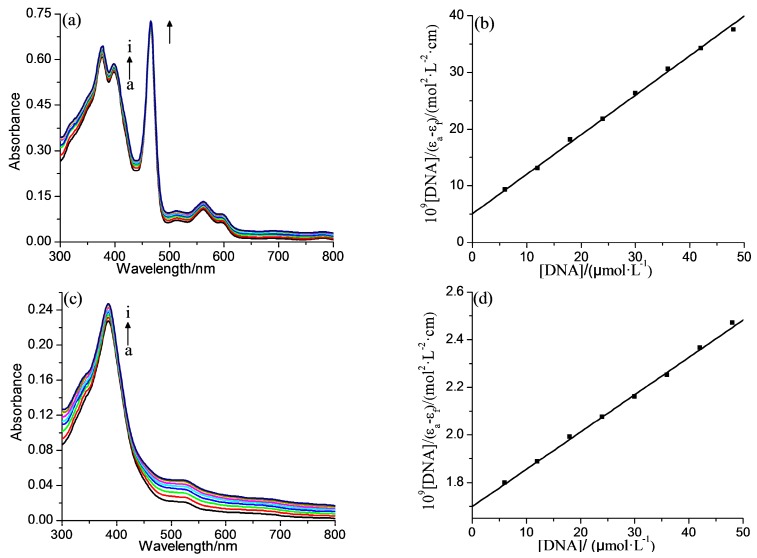
Absorption spectra of (**a**) **2-Mn** (30 μM) and (**c**) **2-Fe** (5 μM) upon the addition of ct-DNA in buffer I at 298 K and pH = 7.2. The arrow shows the intensity changes upon increasing the ct-DNA concentration; (**b**,**d**) are the plots of [DNA]/(*ε*_a_ − *ε*_f_) versus [DNA]. The c_com_ corresponding to 0.0, 6.0, 12.0, 18.0, 24.0, 30.0, 36.0, 42.0, 48.0 (mM) from a to i.

The absorption spectra changes for **2-Mn** and **2-Fe** upon addition of ct-DNA are shown in Figure 3. With the ct-DNA concentration increased, the absorption spectra undergoes a hyperchromic effect. A 6.5% hyperchromism for **2-Mn** and 9.8% for **2-Fe**, without any noticeable spectra shift. The small hyperchromic changes indicate interactions between ct-DNA and both complexes via an outside binding [41].

The intrinsic binding constants (*K*_b_) for the two complexes may be calculated using the following equation [41]:

(1) $$\frac{\left[\mathrm{DNA}\right]}{\varepsilon_{a}-\varepsilon_{f}}=\frac{\left[\mathrm{DNA}\right]}{\varepsilon_{b}-\varepsilon_{f}}+\frac{1}{K_{b}(\varepsilon_{b}-\varepsilon_{f})}$$

where *ε*_a_ is the apparent extinction coefficient of complex in the presence of DNA, *ε*_f_ and *ε*_b_ are the extinction coefficients of the complex when free and fully bound to DNA, respectively; *K*_b_ is the ratio of the slope to the intercept (Figure 2). It turned out 1.31 × 10^5^ M^−1^ and 0.91 × 10^5^ M^−1^ for **2-Mn** and **2-Fe**, respectively. The relatively weaker binding of **2-Fe** with ct-DNA in comparison to **2-Mn** may be attributed to the difference of the coordinated axial between them in the aqueous system. **2-Fe** lost a proton from the coordinated axial water to form [(H_2_O)(OH)Fe^III^P]^4−^, which will increase its negative charge leading the increase of the repulsive force between it and ct-DNA [42]. The ct-DNA binding ability of both complexes were stronger than their free base porphyrin (7.88 × 10^4^ M^−1^), which may be due to the enhancement of the Pi-Pi stacking interaction of metalloporphyrin, and also stronger than TCPP (1.4 × 10^4^ M^−1^) [30], weaker than TSPP (3.74 × 10^5^ M^−1^) [43].

**Figure 4:**
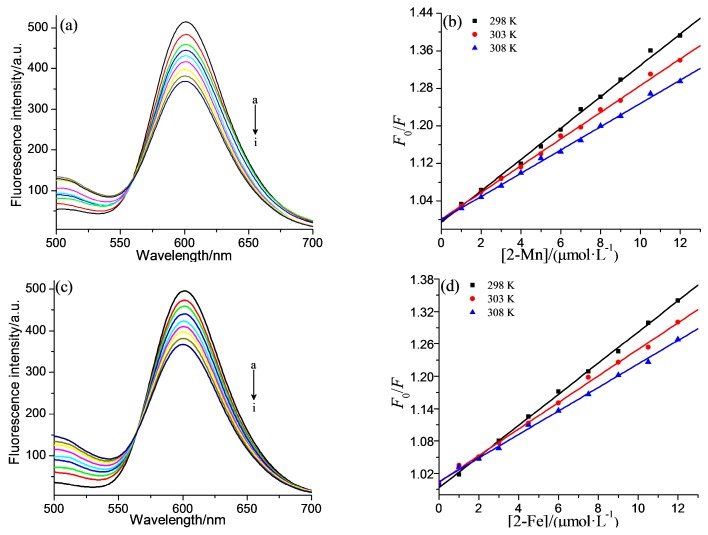
Fluorescence quenching spectra of EB (5 mM) bound to ct-DNA (30 mM) by **2-Mn** (**a**) and **2-Fe** (**c**) in buffer I at 298 K and pH = 7.2. The arrow shows the intensity changes upon increasing the porphyrins concentration; (**b**,**d**) are the plots of *F*_0_/*F* versus [porphyrins] at three different temperatures. The c_com_ corresponding to 0.0, 1.5, 3.0, 4.5, 6.0, 7.5, 9.0, 10.5, 12 (mM) from a to i.

DNA or complex **2-Mn** and **2-Fe** exhibit little fluorescence in aqueous solution, and therefore competitive binding assays were carried out using ethidium bromide (EB) as a probe, to further study the DNA-binding of the complexes. EB emits intense fluorescence when bound to DNA, which could be quenched after the addition of a second DNA-binding molecule by replacing the intercalated EB or by forming a new complex porphyrin-DNA-EB [44,45]. An appreciable decrease in emission intensities was observed upon the addition of **2-Mn** and **2-Fe** to the EB-DNA system (Figure 4). It is known that the binding constant of EB with DNA is 5.16 × 10^5^ L·mol^−1^ [46], so the smaller binding constant between porphyrins and DNA indicates that replacing EB from the complex was impossible. As a result, the second explanation seems more reasonable.

The quenching constants of the system can be analyzed according to the classical Stern-Volmer equation [47]:

*F*_0_/*F* = 1 + *K*_SV_ [Q] = 1 + *K*_q_τ_0_[Q] (2)

where *F*_0_ and *F* are the fluorescence intensities in the absence and presence of complexes, respectively. τ (~10^−8^ s) is the lifetime of the fluorophore and [Q] is the concentration of complex. *K*_SV_ is the liner Stern-Volmer quenching constant which is obtained from the slope of *F*_0_/*F* versus [Q] liner plot, *K*_q_ is the quenching rate constant. The plots of *F*_0_/*F* versus [Q] at three different temperatures (298, 303, 308 K) were displayed in Figure 4. The linear plots suggested that only one type of quenching process occurred. As shown in Table 1, the *K*_sv_ values of both complexes decreased with the increasing temperature and the *K*_q_ values were larger than the limiting diffusion constant of the biomacromolecules (2.0 × 10^10^ L·mol^−1^·s^−1^), which indicated the fluorescence quenching was caused by a static process [30].

**Figure 5:**
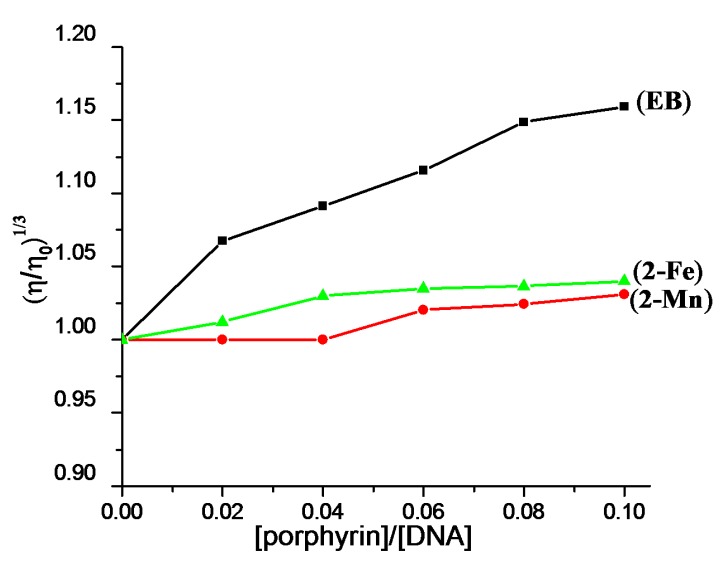
Effect of increasing amounts of the complexes and ethidium bromide on the relative viscosity of ct-DNA (100 mM) in buffer I at 30 ± 0.01 °C and pH = 7.2. [complex]/[DNA] = 0, 0.02, 0.04, 0.06, 0.08, 0.10.

**Figure 6:**
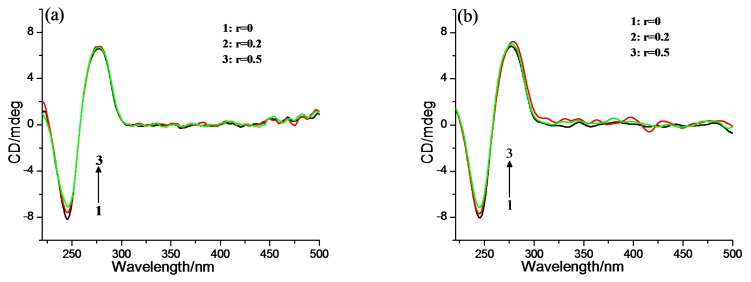
CD spectra of ct-DNA (100 mM) in the absence and presence of (**a**) **2-Mn** and (**b**) **2-Fe** in buffer I at 25 °C and pH = 7.2. *r* = [complex]/[DNA] = 0, 0.2, 0.5.

In order to further check the binding mode between complexes and ct-DNA, viscosity measurement was carried out. The relative viscosity of ct-DNA in the presence of complexes and ethidium bromide is shown in Figure 5. The viscosity of ct-DNA does not change significantly upon continuous addition of **2-Mn** and **2-Fe**, suggesting an outside binding [48]. Figure 6 shows the circular dichroism (CD) spectra changes upon the addition of complexes.

The negative CD peak (at 245 nm) displays little decrease in intensity and the positive band (at 277 nm) remain nearly unperturbed with increasing **2-Mn** and **2-Fe** concentration, which means the binding of **2-Mn** and **2-Fe** to ct-DNA did not change the DNA B-form structure. These observations also imply the binding mode is an outside groove binding [49]. Interestingly, no induced CD at the Soret band for **2-Mn** and **2-Fe** was observed when ct-DNA binds to complexes, which is quite different from carboxyltetraphenyl porphyrin [30]. This indicated the binding mode between **2-Mn** and **2-Fe** and ct-DNA is a random outside binding mode, without the formation of ordered porphyrin aggregate along the DNA minor or major groove [50].

### 2.3. Nuclease Activities

**Figure 7:**
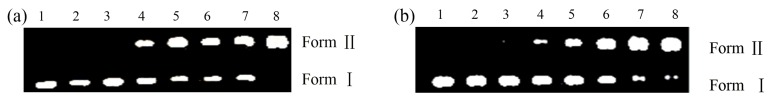
Agarose gel electrophoresis patterns showing the cleavage of supercoiled pBR322 DNA (0.1 mg) by various concentrations of (**a**) **2-Mn** and (**b**) **2-Fe** in the presence of H_2_O_2_ (20 mM) in buffer II (pH = 7.2) for 30 min. lane 1: supercoiled pBR322 DNA alone; lane 2: DNA + H_2_O_2_; lane 3: DNA + porphyrin; lane 4–8: DNA + H_2_O_2_ + 5, 15, 30, 45, 60 μM porphyrin, respectively.

Numerous metal complexes are capable of accelerating DNA cleavage from supercoiled form (form I) to nicked circular form (form II) or linear form (form III) under proper conditions [51,52,53,54]. Iron and manganese porphyrins have been found to cleave DNA in the presence of hydrogen peroxide [55,56,57]. Here, the chemical nuclease activity of manganese and iron porphyrins in the presence of H_2_O_2_ was monitored by gel electrophoresis using pBR322 DNA as target. Figure 7 shows the agarose gel electrophoresis pattern of pBR322 DNA after incubation with **2-Mn** and **2-Fe**. No DNA cleavage could be observed for pure DNA (lane 1), DNA with oxidant (lane 2) or complexes (lane 3) alone. While DNA upon exposure to a solution containing both complexes and H_2_O_2_ (lanes 4–8), supercoiled pBR322 DNA underwent remarkable cleavage from supercoiled form (form I) to nicked circular form (form II). The oxidative DNA cleavage significantly depends on the concentration of complexes. Nearly all the supercoiled DNA was consumed when the concentration of **2-Mn** reached 60 μM, while 90% supercoiled DNA was consumed when using **2-Fe** at the same conditions.

**Figure 8:**
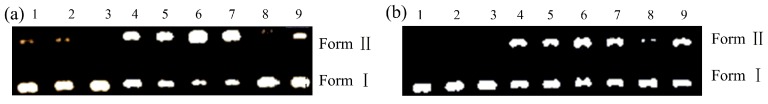
Agarose gel electrophoresis patterns showing the cleavage of supercoiled pBR 322 DNA (0.1 mg) by (**a**) **2-Mn** and (**b**) **2-Fe** in the presence of H_2_O_2_ and additives in buffer II (pH = 7.2) for 30 min. lane 1: supercoiled pBR322 DNA alone; lane 2: DNA + 20 mM H_2_O_2_; lane 3: DNA + 20 μM porphyrin; lanes 4–9: DNA + 20 mM H_2_O_2_ + 20 μM porphyrin + 0, NaN_3_ (50 mM), DABCO (50 mM), l-histidine (50 mM), DMSO (50 mM), *tert*-BuOH (50 mM), respectively.

To investigate the possible reactive oxygen species (ROS) for pBR322 DNA cleavage in the present system, inhibiter tests were carried out and the results are shown in Figure 8. In the presence of hydroxyl radical (·OH) scavenger DMSO (dimethyl sulfoxide) (lane 8) or *tert*-butyl alcohol (TBA) (lane 9), the cleavage was inhibited significantly, indicating the possible involvement of the hydroxyl radical. The addition of singlet oxygen (^1^O_2_) quenchers NaN_3_ (lane 5), DABCO (lane 6) or L-histidine (lane 7) has no effect on DNA cleavage. It rules out the involvement of singlet oxygen when using H_2_O_2_ as oxidant for DNA cleavage by both complexes.

As porphyrins were unstable under oxidation conditions due to their self-destruction, the stability of **2-Mn** and **2-Fe** in the presence of H_2_O_2_ with the ratio of *r* is 666 (*r* = H_2_O_2_/porphyrin) was investigated by UV-Vis spectroscopy. As shown in Figure S13, **2-Mn** was decomposed about 30% in 30 min, while **2-Fe** was nearly decomposed totally. Thus, they are most possibly being destroyed by the ROS.

### 2.4. Cytotoxicity

The in vitro inhibitory activities of **2**, **2-Mn** and **2-Fe** against human breast cells (MCF-7), human hepatocellular carcinoma cells (Hep G2) and human cervical carcinoma cells (HeLa) were evaluated by an MTT assay. As shown in Table 2, **2-Mn** exhibited no dark cytotoxicity to any of the test cell lines. Under light irradiation, there is no big improvement in the cytotoxicity of **2-Fe**. Interestingly, the cytotoxicity of **2-Mn** to Hep G2 cell lines was sharply increased under light irradiation. These observations suggest the cytotoxicity of metal *meso*-tetrakis (carboxyl) porphyrins depend largely not only on the central metals, but also the type of tumor cell lines [58]. The phototoxicity of *m*THPC was also obtained under the same experimental conditions, as a positive control. The activity of *m*THPC is much better than all the test compounds. Besides, **2-Mn** was found to be less phototoxicity on the healthy cell line (BEAS-2B) than the cancer cells in this work. The enhanced photocytotoxicity relative to the dark for **2-Mn** is an essential property of a photochemotherapeutic agent. Although the phototoxicity of **2-Mn** was rather lower than the positive control, **2-Mn** was found to be less phototoxicity on the healthy cell line (BEAS-2B) than the cancer cells in this work, which is suggestive of a better therapeutic profile. Besides, **2-Mn** shown more better phototoxicity against Hep G2 cells (ratios −/+*hv* for the IC_50_ values) compare with the other lings in this work.

**Figure 9:**
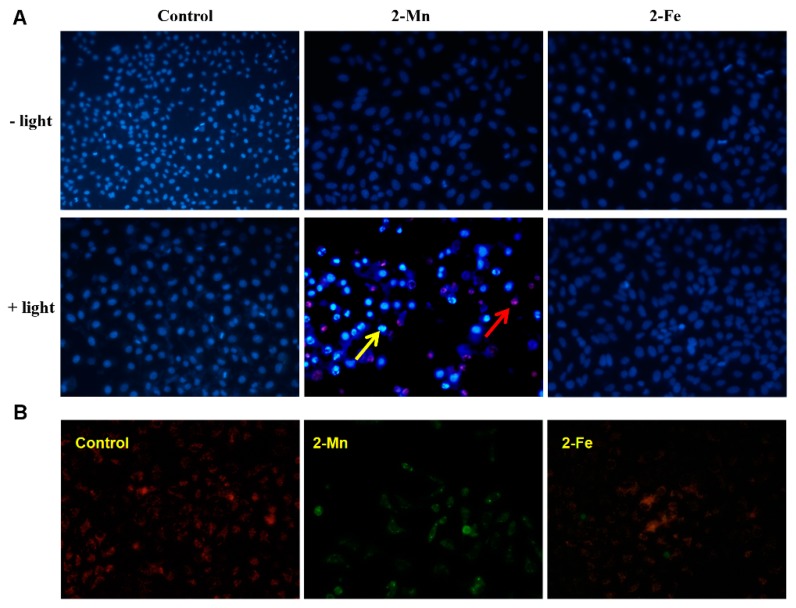
(**A**) Fluorescence microscopic images of Hoechst-33342 and PI stained Hep G2 cells after treatment with **2-Mn** (20 μM) and **2-Fe** (20 μM). Red arrow the early apoptosis cell, yellow arrow the late apoptosis cell; (**B**) Effect of **2-Mn** (20 μM) and **2-Fe** (20 μM) on the MMP decrease in Hep G2 cells.

As shown in Figure 9, the morphological changes of Hep G2 cells in the absence and presence of **2-Mn** and **2-Fe** were further investigated. In the phase-contrast observation, the Hep G2 cells incubated with **2-Mn** and irradiation displayed a reduction in cell number, cell shrinkage and loss of cell-to-cell contact. The reduction of cell viability and the change in cell morphology further proved the growth inhibitory effect of **2-Mn** on Hep G2 cells. Cells receiving either treatment alone showed no such phenotype. This indicates that light at specific wavelength scan promote **2-Mn** cytotoxicity to cancer cells [58]. Such morphological changes are often observed in apoptotic and necrosis cells, and thus **2-Mn** may induce cell death in Hep G2 cells through an apoptotic/necrosis pathway. However, Hep G2 cells receiving **2-Fe** treatment showed no such phenotype.

**Figure 10:**
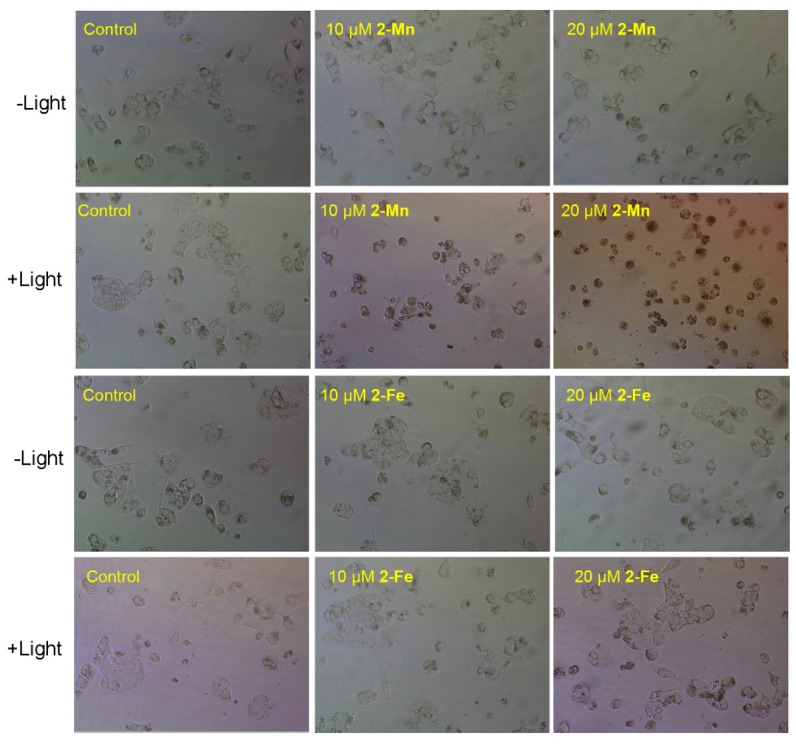
The morphological changes of human hepatocellular carcinoma cells in the absence and presence of **2-Mn** and **2-Fe** with/without light.

To investigate the mechanism of irradiation enhanced cytotoxicity of **2-Mn**, nuclear staining was firstly performed in the Hep G2 cells with/without irradiation. In normal nucleus, the extended euchromatin is visible in blue and showed no remarkable changes under fluorescence microscope after staining with Hoechst-33342/PI (Figure 10A). After treatment with **2-Mn** in dark, there are no obvious changes in the density and morphological images of cells. This suggests the cells were resistant to **2-Mn** treatment without irradiation. After receiving **2-Mn** and light, however, not only did massive bright spots appear (a typical apoptotic feature) and the density of the cells be reduced, but also morphological changes of the nucleus were also observed [58], such as condensed chromatin and fragmented nuclei (red arrow). Cells receiving either treatment alone showed no such phenotype. The PDT therapy of **2-Mn** also caused the Hep G2 cells to display late apoptotic features (yellow arrow). Such morphological changes and bright staining of the nucleus are often observed in apoptotic cells, and thus **2-Mn** may induce cell death in Hep G2 cells through an apoptotic pathway.

**Figure 11:**
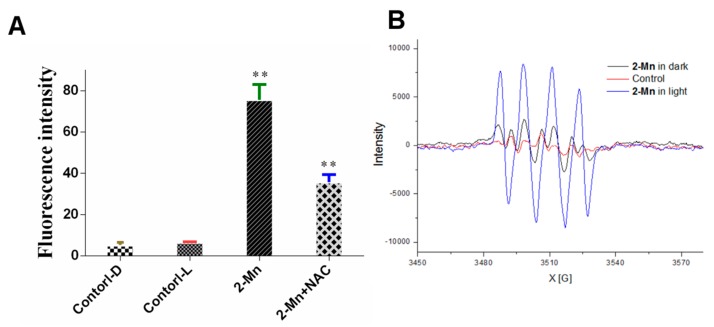
(**A**) Intracellular ROS detection in Hep G2 cells after treatment with **2-Mn** by DCFH-DA, NAC (a free radical scavenger) with/without light, ** *p* < 0.01, significantly different compared with the control by *t*-test, n = 3; (**B**) Superoxide and hydroxyl radical detection of **2-Mn** with/without light by EPR.

Mitochondria play an essential role in the progression of apoptosis. Decreased MMP has been implicated as a typical event of apoptotic cells [59]. When the cell is in an apoptotic state, the MMP is reduced and the fluorescence of JC-1 will change from red to green. The results of fluorescence microscopic (Figure 10B) indicated that the photocytotoxicity of **2-Mn** can induce the decrease of MMP and cause mitochondrial dysfunction. MMP collapse is closely associated with the mitochondrial production of ROS [60], which plays an important role in cell apoptosis. Therefore, we further evaluated the intra-cellular ROS generation. As shown in Figure 11A, the results demonstrated that the ROS play a key role in the Hep G2 cell apoptosis process by **2-Mn** photocytotoxicity. NAC, a free radical scavenger, which could block the **2-Mn** photocytotoxicity induced production of ROS was also found (Figure 11A). Hence, we tentatively conclude that **2-Mn** photocytotoxicity caused Hep G2 cell apoptosis by inducing ROS production and activating the mitochondrial damage pathway. To investigate ROS production of **2-Mn** under irradiation, an EPR spectrum was added, which can reflect the change of superoxide and hydroxyl radical (one kind of ROS). When superoxide and hydroxyl radical was produced, quartet/sextet can be found. As shown in Figure 10B, in the control group, no signal was found in the EPR spectrum. However, after treatment with **2-Mn** under light, a typical quartet was obtained, and the intensity increased dozens of times compare with **2-Mn** under dark, suggesting superoxide and hydroxyl radical was produced [52]. The detailed mechanism was still needed to be further investigated.

## 3. Experimental Section

### 3.1. General Information

All reagents and chemicals, purchased from commercial sources, were of analytical grade and used without further purification. Calf thymus DNA (ct-DNA) were obtained from Sigma-Aldrich (Shanghai, China). Tris (hydroxymethy) aminomethane (Tris-base), ethidium bromide (EB), sodium chloride, boric acid (H_3_BO_3_), dimethyl sulfoxide (DMSO), 30% hydrogen peroxide (H_2_O_2_), agarose gel loading buffer, and ethylenediaminetetraacetic acid (EDTA), pBR322 DNA were purchased from Shanghai Sangon Company (Shanghai, China) and were of biological grade. All aqueous solutions were prepared from deionized water. Buffer I, 5 mM Tris-HCl/50 mM NaCl in deionized water (pH = 7.2), was used for preparing stock solution of ct-DNA, absorption titrations, fluorescence studies, viscosity experiments and circular dichroism detecting. Buffer II, 50 mM Tris-HCl/18 mM NaCl in deionized water (pH = 7.2), Buffer III, Tris-boric acid-EDTA (89 mM Tris, 89 mM H_3_BO_3_, 20 mM EDTA, pH = 8.3) in aqueous solution, both two buffer solutions were used for gel electrophoresis experiments. MTT, pancreatin, Hoechst 33342 and PI were purchased from Sigma-Aldrich. Dulbecco’s modified eagle medium (DMEM) and bovine serum were obtained from Life Technologies (Shanghai, China) and Zhejiang Tianhang Biological Technology Stock Co., Ltd. (Hangzhou, China), respectively. JC-1 kit and DCFH-DA kit were get from Beyotime Biotechnology (Beijing, China). The ct-DNA stock solution was prepared in buffer I and its concentration was determined by measuring the absorption intensity at 260 nm with a molar extinction coefficient value of 6600 M^−1^cm^−1^ [61].

UV-Vis absorption spectra were measured on 3900H UV-Vis spectrometer (Hitachi, Tokyo, Japan) in quartz cells with a 1.0 cm optical path length at room temperature. Emission spectra were recorded on a RF-5301 fluorescence spectrophotometer (Perkin Elmer, Los Angeles, CA, USA) with a THX-05 water bath and 1.0 cm quartz cell at three different temperatures. The circular dichroism (CD) spectra were monitored on a J-810 spectrometer (JASCO, Tokyo, Japan). The viscosity experiments were carried out by using an Ubbelodhe viscometer. The agarose gel electrophoresis was conducted on a DYCP-31CN electrophoresis cell (Liuyi, Beijing, China), and then analyzed by using a Gel Documentation System (Bio-Rad, Beijing, China). ^1^H-NMR spectra were recorded on an Avance 400 MHz spectrometer (Bruker, Karlsruhe, Germany) in CDCl_3_ solution. HR-MS spectra were recorded on a Bruker maXis impact mass spectrometer with an ESI source by using α-cyano-4-hydroxycinnamic acid (HCCA) as matrix. The cells were observed with an EVOS XL Core Life stereomicroscope (Life technologies EVOS^®^, Thermo, MA, USA).

### 3.2. Synthesis of ***2-Mn*** and ***2-Fe***

*5,10,15,20-Tetrakis(ethoxycarbonyl)porphyrin* (**1**). In a 1-L round-bottom flask equipped with a mechanical stirrer, a solution of ethyl glyoxylate in toluene (50 %, (1.33 mL, 6.5 mmol), freshly distilled pyrrole (0.468 mL, 6.7 mmol) and CH_2_Cl_2_ (DCM, 500 mL, stabilized by ethanol) were added. The reaction mixture was stirred for 5 min, then BF_3_⋅Et_2_O (0.2 mL, 1.6 mmol) was added. After stirring for a period of 100 min at room temperature, the reaction was quenched by triethylamine (1.0 mL) and followed by the addition of 2,3-dichloro-5,6-dicyano-1,4-benzoquinone (DDQ, 1.48 g, 6.5 mmol). The reaction mixture was stirred for an additional 40 min. The reaction mixture was poured onto a short silica gel column to run a flash chromatography separation, and the solvent of the collected fractions was removed by rotary evaporation to afford the crude product, which was further purified by chromatography on silica gel with CH_2_Cl_2_/hexanes (4:1) as eluent. The pure product was obtained after recrystallization from CH_2_Cl_2_/hexane. Yield: 8.0%. *R*_f_ = 0.57 (CH_2_Cl_2_/Hexanes: 4/1); UV-Vis (CH_2_Cl_2_) *λ*_max_(log ε): 407 (5.33), 505 (4.20), 540 (3.59), 583 (3.75), 638 (3.44) nm; ^1^H-NMR (CDCl_3_) δ: 9.54 (s, 8H), 5.12 (q, *J* = 7.1 Hz, 8H), 1.82 (t, *J* = 7.1 Hz, 12H), -3.35 (s, 2H); ^13^C-NMR (CDCl_3_) δ: 170.48, 131.43, 112.18, 63.49, 14.76; Elemental Analysis, anal. calcd. for C_32_H_31_N_4_O_8_: C, 64.21%; H, 5.05%; N, 9.36%, found C, 64.11%; H, 5.00%; N, 9.46%; HR-MS, calcd. for C_32_H_31_N_4_O_8_: 599.2136, found *m*/*z*: 599.2142 [M + H]^+^.

*5,10,15,20-Tetrakis(ethoxycarbonyl)porphyrin manganese* (III) (**1-Mn**). A solution of 5,10,15,20-tetrakis (ethoxycarbonyl) porphyrin (59.8 mg, 0.1 mmol) and manganese acetate tetrahydrate (245 mg, 1 mmol) in *N*,*N*-dimethylformamide (DMF, 30 mL) was refluxed for 4 h. The reaction mixture was allowed to cool down to room temperature, then CH_2_Cl_2_ (30 mL) and H_2_O (30 mL) were added to the reaction mixture, followed by stirring vigorously for 5 min. The organic phase was collected and washed six times with deionized water. The resulting crude product was purified by chromatography on silica gel with CH_2_Cl_2_/CH_3_OH (100:5) as eluent. The pure product was obtained after recrystallization from CH_2_Cl_2_/hexane. Yield: 95.0%. *R*_f_ = 0.22 (CH_2_Cl_2_/CH_3_OH: 95/5); UV-Vis (CH_2_Cl_2_) *λ*_max_ (log ε): 364 (4.80), 474 (4.90), 570 (4.07) nm; Elemental Analysis, anal. calcd. for C_32_H_28_N_4_O_8_Mn: C, 55.95%; H, 4.11%; N, 8.16%, found: C, 55.97%; H, 4.10%; N, 8.20%; HR-MS, calcd. for C_32_H_28_N_4_O_8_Mn: 651.1282, found *m*/*z*: 651.1285 [M − Cl]^+^.

*5,10,15,20-Tetrakis(ethoxycarbonyl)porphyrin iron* (III) (**1-Fe**). **1-Fe** was synthesized by a similar procedure as **1-Mn**, excepting iron chloride tetrahydrate (200 mg, 1 mmol) was used instead of manganese acetate tetrahydrate and the reaction mixture was washed with concentrated HCl. Yield: 90.0%. *R*_f_ = 0.28 (CH_2_Cl_2_/CH_3_OH: 95/5); UV-Vis (CH_2_Cl_2_) *λ*_max_(log ε): 361 (4.55), 409 (4.76), 509 (3.76), 635 (3.45) nm; Elemental Analysis, anal. calcd. for C_32_H_28_N_4_O_8_Fe: C, 55.87%; H, 4.10%; N, 8.14%, found: C, 55.84%; H, 4.02%; N, 8.20%; HR-MS, calcd. for C_32_H_28_N_4_O_8_Fe: 652.1251, found *m*/*z*: 652.1248 [M − Cl]^+^.

*5,10,15,20-Tetrakis(carboxyl)porphyrin* (**2**). The obtained **1** (22 mg) was dissolved in a mixed solvent of THF/CH_3_OH (5:2, 126 mL) and 0.5 M aq. KOH (38 mL). The mixture was stirred for 24 h at room temperature. The reaction was monitored by thin layer chromatography using CH_2_Cl_2_/CH_3_OH (100:5) as eluent. After all starting material **1** was consumed, the reaction mixture was acidized with HCl (5% aq). The resulting product was extracted with THF/CH_2_Cl_2_ (1:1) mixed solvent, the organic phase was collected and washed with water for three times, dried over Na_2_SO_4_, filtered and evaporated under reduced pressure. The pure product was obtained after recrystallization from acetone/hexane. Yield: 85.0%. *R*_f_ = 0.10 (CH_3_OH); UV-Vis (Buffer I) *λ*_max_(log ε): 406 (5.27), 508 (3.91), 537 (3.49), 578(3.35), 632(3.34) nm; ^1^H-NMR (DMSO-*d*_6_) δ: 9.58 (s, 8H), −3.48 (s, 2H); Elemental Analysis, anal. calcd. for C_24_H_15_N_4_O_8_: C, 59.27%; H, 2.90%; N, 11.52%, found: C, 59.26%; H, 2.91%; N, 11.51%; HR-MS, calcd. for C_24_H_15_N_4_O_8_: 487.0890; found *m*/*z*: 487.0886 [M + H]^+^.

*5,10,15,20-Tetrakis(carboxyl)porphyrin manganese* (III) (**2-Mn**). The obtained **1-Mn** (25 mg) was dissolved in a mixed solvent of THF/CH_3_OH (5:2, 126 mL) and 0.5 M aq. KOH (38 mL). The mixture was stirred for 24 h at room temperature. The reaction was monitored by thin layer chromatography using CH_2_Cl_2_/CH_3_OH (100:5) as eluent. After all starting material **1-Mn** was consumed, the reaction mixture was acidized with HCl (5% aq). The resulting product was extracted with THF/CH_2_Cl_2_ (1:1) mixed solvent, the organic phase was collected and washed with water for three times, dried over Na_2_SO_4_, filtered and evaporated under reduced pressure. The pure product was obtained after recrystallization from acetone/hexane. Yield: 85.0%. *R*_f_ = 0.10 (CH_3_OH); UV-Vis (Buffer I) *λ*_max_(log ε): 376 (4.31), 398 (4.27), 466 (4.375), 562 (3.57), 599(3.35) nm; Elemental Analysis, anal. calcd. for C_24_H_12_N_4_O_8_Mn: C, 50.15%; H, 2.10%; N, 9.75%, found: C, 50.17%; H, 2.02%; N, 9.85%; MALDI-TOF: *m*/*z* calcd. for C_24_H_12_N_4_O_8_Mn: 539.312; found: 538.847 [M]^+^.

*5,10,15,20-Tetrakis (carboxyl) porphyrin iron* (III) (**2-Fe**). **2-Fe** was synthesized by a similar procedure as **2-Mn**, Yield: 80.0%. *R*_f_ = 0.15 (CH_3_OH); UV-Vis (Buffer I) *λ*_max_(log ε): 389 (4.64), 510(3.46), 630 (3.01) nm; Elemental Analysis, anal. calcd. for C_24_H_12_N_4_O_8_Fe: C, 50.07%; H, 2.10%; N, 9.73%, found: C, 50.10%; H, 2.15%; N, 9.75%; MALDI-TOF: *m*/*z* calcd. for C_24_H_12_N_4_O_8_Fe: 540.000; found: 539.979 [M]^+^.

### 3.3. DNA Binding Experiments

#### 3.3.1. UV Spectroscopy

Electronic absorption spectra titrations were monitored at the range of 300–800 nm during gradual addition of ct-DNA (0~54 mL) to the samples in buffer I at room temperature. The concentration of ct-DNA stock solution was 3 mM. Absorption values were recorded after each successive addition of 6 mL ct-DNA and incubation for 5 min.

#### 3.3.2. Fluorescence Spectroscopy

Emission spectra were recorded by increasing the concentration of the sample (0~24 mL ) in 3 mL of a solution of ethidium bromide (EB) plus ct-DNA in buffer I with the excitation wavelength set at 370 nm. The changes in emission spectra were recorded after each successive addition of 3 mL sample and incubated for 5 min in the range of 500–700 nm at different temperatures. The concentration of sample stock solution is 1.5 mM.

#### 3.3.3. CD Spectroscopy

CD spectra of ct-DNA (100 mM) in the absence and presence of samples (*r* = 0, 0.20, 0.50, where *r* is the molar ratio of complex to ct-DNA) were determined in buffer I at 25 °C with a 1.0 cm quartz cell. The CD spectra were run from 600 to 220 nm and the buffer background was automatically subtracted. All CD spectra were generated after averaging three scans. The CD spectra of ct-DNA alone were recorded as the control experiment.

#### 3.3.4. Viscosity Experiments

The viscosity of ct-DNA (100 mM) in the absence and presence of complexes (*r* = 0, 0.02, 0.04, 0.05, 0.06, 0.08, 0.10, where *r* is the molar ratio of complex to ct-DNA) in buffer I were performed using an Ubbelohde Viscometer at 30.0 ± 0.01 °C in a thermostatic bath. Each sample was measured five times and an average flow time was determined. Data were presented as (η/η_0_)^1/3^ versus the concentration ratio of complex to ct-DNA, where η_0_ was the relative viscosities of ct-DNA alone and η was the viscosities of ct-DNA in the presence of complex, respectively. The control sample was carried out with EB by using the same method.

### 3.4. DNA Cleavage Experiments

Cleavage activity of the complexes was monitored using agarose gel electrophoresis experiments performed as follows: the 10 μL mixture of pBR 322 DNA (0.1 mg), oxidants and variable concentration of complexes were incubated in the dark for 30 min and then the loading buffer (2 μL) was added to quench the reactions. The resulting solutions were analyzed by electrophoresis for 2 h at 70 V on 1.1% agarose gel in TBE buffer. Finally the gel was stained with 1.0 mg·L^−1^ EB solution and photographed with a Gel Doc XR system (Bio-Rad).

### 3.5. Cytotoxic Activity Assay

Human cancer cell lines were purchased from American Type Culture Collection (ATCC, Manassas, VA, USA), including MCF-7, Hep G2, HeLa and BEAS-2B. All cell lines were maintained in DMEM media supplemented with fetal bovine serum (10%) at 37 °C in CO_2_ incubator (95% relative humidity, 5% CO_2_). The tested compounds were dissolved in Phosphate Buffer Solution (PBS) at 10 mM. Cell viability was determined by measuring the ability of cells to transform MTT to a purple formazan dye. Cells were seeded in 96-well tissue culture plates (5 × 10^3^ cells/well) for 24 h. To investigate the photodynamic potential of the target complexes, we incubated the cells with increasing concentrations of tested compounds (0–320 μM), after PDT treatment (LED, 625 mn, 5 W, red light, 60 min, 15.5 cm from the light), the cells were incubated with the tested compounds for 23 h. Cells incubating with the tested compounds without illumination (dark control) were kept in parallel. After incubation, 20 μL/well of MTT solution (5.0 mg/mL phosphate buffered saline) was added and incubated for 5 h. The medium was aspirated and replaced with 150 μL/well DMSO to dissolve the formazan salt. The absorbance intensity, which reflects the cell growth condition, was measured at 570 nm using a microplate spectrophotometer (Versamax, Thermo, Waltham, MA, USA). The morphological changes of Hep G2 cells were also tested by the stereomicroscope (EVOS XL Core Life). Cells were seeded in 6-well tissue culture plates (10^5^ cells/well) for 12 h, then we incubated the cells with **2-Mn** and **2-Fe** as previously described. The morphological images were obtained by stereomicroscope without other treatment. The same procedure was carried out without light irradiation for determining dark toxicity.

### 3.6. Nuclear Staining

Hep G2 cells were firstly seeded (2 × 10^5^ per well) on a six-well plate the day before PDT treatment, then treated with **2-Mn** and **2-Fe** under light as previously described. Untreated cells were used as control. Cell apoptosis was evaluated by in situ uptake of Hoechst 33342 (5 μg/mL) as described previously, and visualized using a fluorescence microscope (TE2000-E, Nikon, Tokyo, Japan).

### 3.7. Change of MMP

Hep G2 Cells were seeded in 6-well tissue culture plates (10^6^ cells/well) for 12 h, then the cells were added **2-Mn**, after illuminated, the cells were incubated with **2-Mn** further 23 h. After washed three times with cold PBS, cells were incubated for 20 min with 1 mL JC-1 in culture medium at 37 °C in the dark for 20 min. After washed three times with JC-1 buffer, the cells were detached with pancreatin solution. Collected Cells were immediately centrifuged to remove the supernatant, and then visualized by fluorescence microscopy (TE2000-E, Nikon).

### 3.8. ROS Levels Assay

Hep G2 Cells were seeded in 6-well tissue culture plates (10^6^ cells/well) for 12 h, then the cells were added **2-Mn**, after illuminated the cells were incubated with **2-Mn** further 23 h. NAC (5 mM) group was firstly incubated 2 h before treatment with **2-Mn** and irradiation. After incubation 6 h, the fluorescent dye DCFH-DA was added to the medium with anal concentration of 10 μM to cover the cells. After 30 min in the dark, the treated cells were then washed with DMEM, collected by trypsinization and centrifugation at 1500 rpm for 5 min, and resuspended in PBS. Fluorescence intensity was determined by a microplate analyzer (Varioskan Flash, Thermo, Waltham, MA, USA) with an excitation wavelength of 488 nm and emission at 525 nm. Data are presented as the mean ± standard error of the mean, *n* = 3.

### 3.9. The EPR Spectra

The EPR spectra were taken at room temperature on a Bruker ESP-300E spectrometer at 9.8 GHz, X-band with 100 Hz field modulation. Samples were injected quantitatively into quartz capillaries and illuminated in the cavity of the EPR spectrometer with a Nd:YAG laser at 532 nm (5–6 ns of pulse width, 10 Hz of repetition frequency, 30 mJ/pulse energy).

### 3.10. Statistical Analysis

Data are presented as the means ± standard error of the mean (SEM) from three independent experiments. Statistical analysis was performed using one-way analysis of variance (ANOVA) followed by a Bonferroni post-hoc for multiple group comparison or Student’s unpaired *t*-test for two-group comparison where appropriate. The analyses were performed using GraphPad Prism Software version 6.0 (GraphPad Inc., La Jolla, CA, USA).

## 4. Conclusions

In conclusion, two anionic metalloporphyrins, manganese porphyrin (**2-Mn**) and iron porphyrin (**2-Fe**), were synthesized and characterized. Both metalloporphyrins exhibit good affinity to ct-DNA and the binding constant is on the order of about 10^4^~10^5^ M^−1^. Spectroscopic and viscosity experimental results showed the interaction between both complexes and ct-DNA is via an outside binding mode. Both complexes are efficient DNA cleavage agents in the presence of H_2_O_2_. **2-Mn** exhibits considerable photocytotoxicity against Hep G2 cells by triggering a significant generation of ROS.

## Figures and Tables

**Table 1:** Stern-Volmer quenching constants for the interaction of **2-Mn** and **2-Fe** with ct-DNA at three different temperatures and pH = 7.2.

Compound	*T*/K	K_sv_/L·mol^−1^	K_q_/L·mol^−1^·s^−1^	R
**2-Mn**	298	3.33 × 10^4^	3.35 × 10^12^	0.9977
	303	2.84 × 10^4^	2.85 × 10^12^	0.9985
	308	2.48× 10^4^	2.48 × 10^12^	0.9986
**2-Fe**	298	2.89 × 10^4^	2.89 × 10^12^	0.9990
	303	2.46× 10^4^	2.46 × 10^12^	0.9979
	308	2.18 × 10^4^	2.18 × 10^12^	0.9980

**Table 2:** The cytotoxic activity of **2**, **2-Mn** and **2-Fe** against selected cell lines in the same conditions.

Compound	IC_50_ Value /(μM/L) (Dark)				IC_50_ Value/(μM/L) (Light)			
	MCF-7	Hep G2	Hela	BEAS-2B	MCF-7	Hep G2	Hela	BEAS-2B
**2**	174.4 ± 4.9	306.7 ± 36.4	>320	>320	>320	49.1 ± 13.8	74.9 ± 19.6	89.4 ± 15.2
**2-Mn**	>320	100.5 ± 4.7	61.7 ± 3.9	226.2 ± 15.7	>320	19.0 ± 2.5	54.2 ± 4.2	245.3 ± 13.9
**2-Fe**	>320	175.5 ± 7.4	>320	>320	139.9 ± 7.7	137.5 ± 8.9	>320	>320
***m*THPC**	177.1 ± 19.5	129.3 ± 16.2	168.3 ± 24.9	180.5 ± 17.4	1.1 ± 0.3	1.3 ± 0.5	1.6 ± 0.8	1.9 ± 0.8

The activity of *meta*-tetrahydroxyphenyl chlorine (*m*THPC, Foscan) was also obtained under the same experimental conditions.

## Supplementary Materials

Supplementary materials are available online.
