# Supplementary material for: Water-soluble Manganese and Iron Mesotetrakis(carboxyl)porphyrin: DNA Binding, Oxidative Cleavage, and Cytotoxic Activities
Source: Molecules. 2017 Jun 29;22(7):1084. doi: 10.3390/molecules22071084 (PMC6152243; doi:10.3390/molecules22071084)
Supplement: Supplementary file 1 [file molecules-22-01084-s001.pdf]

Supporting information for

Water-soluble Manganese and Iron *meso*-tetrakis(carboxyl)porphyrin: DNA Binding, Oxidative Cleavage, and Cytotoxic Activities

Lei Shi [1,2,*], Yi-Yu Jiang [3], Tao Jiang [1], Wei Yin [1,2], Jian-Ping Yang [1,2], Man-Li Cao [1,2], Yu-Qi Fang [1] and Hai-Yang Liu [3,*]

1 Department of Chemistry, Guangdong University of Education, Guangzhou, 510303, China, shil@gdei.edu.cn(L.S.); jt@gdei.edu.cn (T.J.); yinwei@gdei.edu.cn (W.Y.); yangjianping@gdei.edu.cn (J.-P.Y.); caomanli@gdei.edu.cn (M.-L.C.); 15622197246@163.com (Y.-Q.F.);

2 Engineering Technology Development Center of Advanced Materials & Energy Saving and Emission Reduction in Guangdong Colleges and Universities, Guangzhou 510303, China

3 Department of Chemistry, South China University of Technology, Guangzhou, 510641, China; 529117636@qq.com (Y.-Y.J.); chhyliu@scut.edu.cn (H.-Y.L.)

* Correspondence: shil@gdei.edu.cn(L.S.); chhyliu@scut.edu.cn (H.-Y.L.)
Tel.: +86-20-3411-3456 (L.S.); +86-20-2223-6805 (H.-Y.L.)

Table of Contents

1. Characterization of compounds..........2

2. HPLC trace of porphyrins 2, 2-Mn and 2-Fe..........10

3. The UV-Vis spectra changes of 2-Mn (a) and 2-Fe (b) upon the addition H_2O_2..........11

1. Characterization of compounds

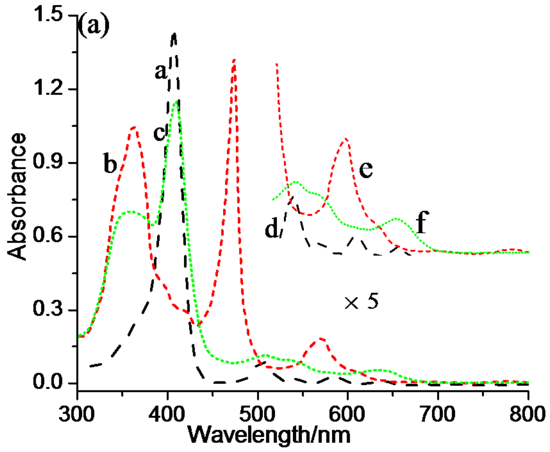

Figure S1. UV-Vis absorption spectra of **1**, **1-Mn** and **1-Fe** in dichloromethane.

a: **1**-Soret band; **b**: **1-Mn**-Soret band; **c**: **1-Fe**-Soret band; **d**: **1**-Q band; **e**: **1-Mn**-Q band; **f**: **1-Fe**-Q band

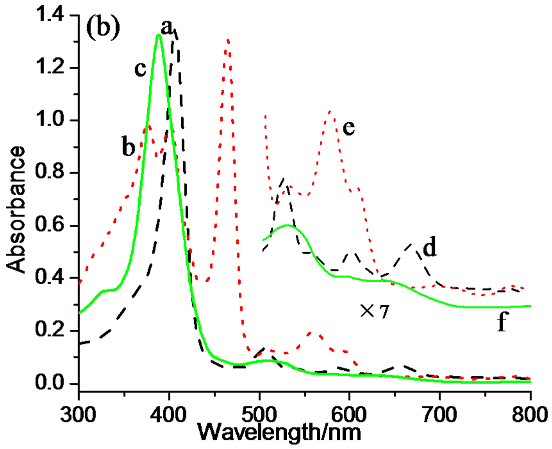

Figure S2. UV-Vis absorption spectra of **2**, **2-Mn** and **2-Fe** in 5 mM Tris-HCl/50 mM NaCl buffer.

a: **2**-Soret band; **b**: **2-Mn**-Soret band; **c**: **2-Fe**-Soret band; **d**: **2**-Q band; **e**: **2-Mn**-Q band; **f**: **2-Fe**-Q band

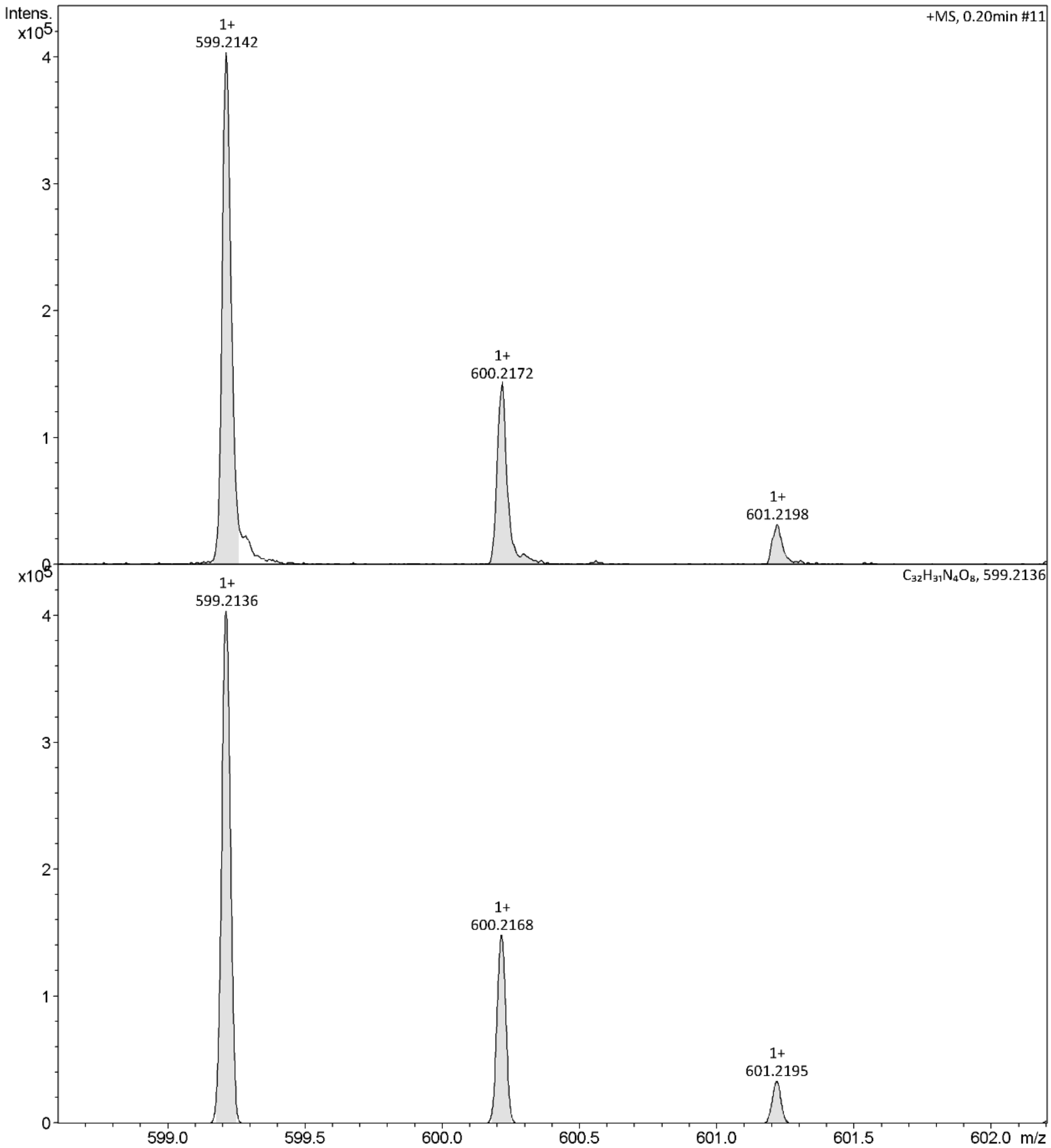

Figure S3. HR-MS of 5, 10, 15, 20-tetrakis(ethoxycarbonyl)porphyrin (**1**)

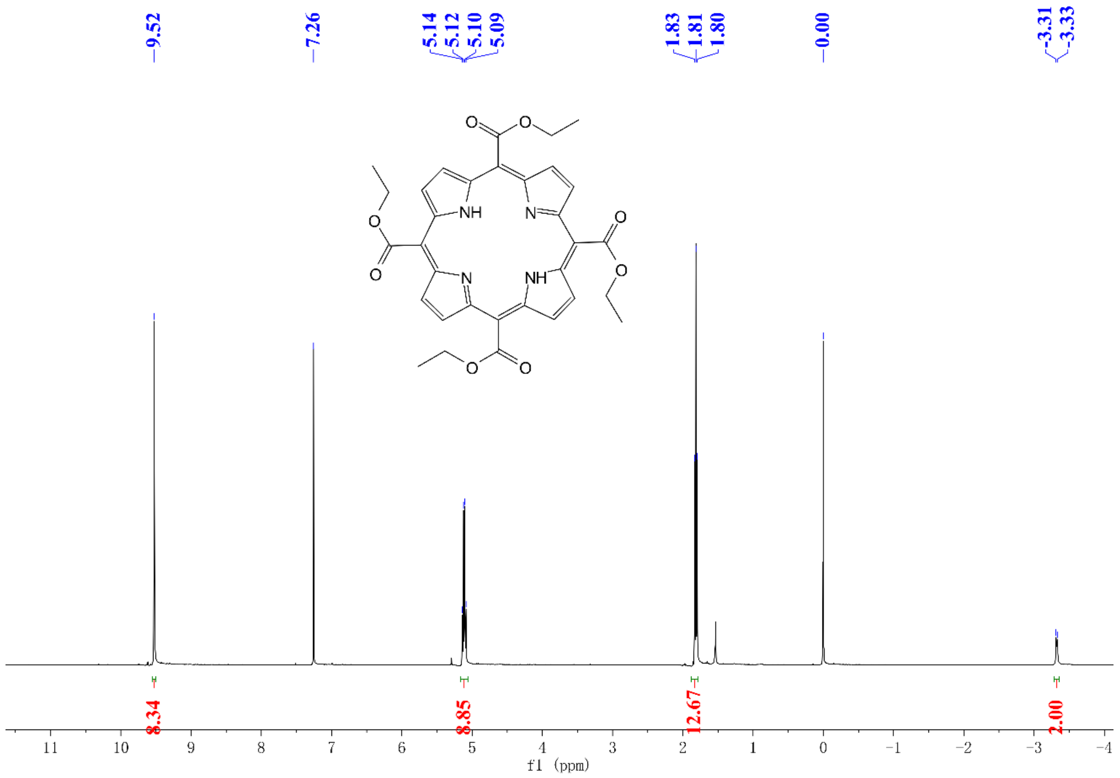

Figure S4. ^{1}H NMR of 5, 10, 15, 20-tetrakis(ethoxycarbonyl)porphyrin (**1**).

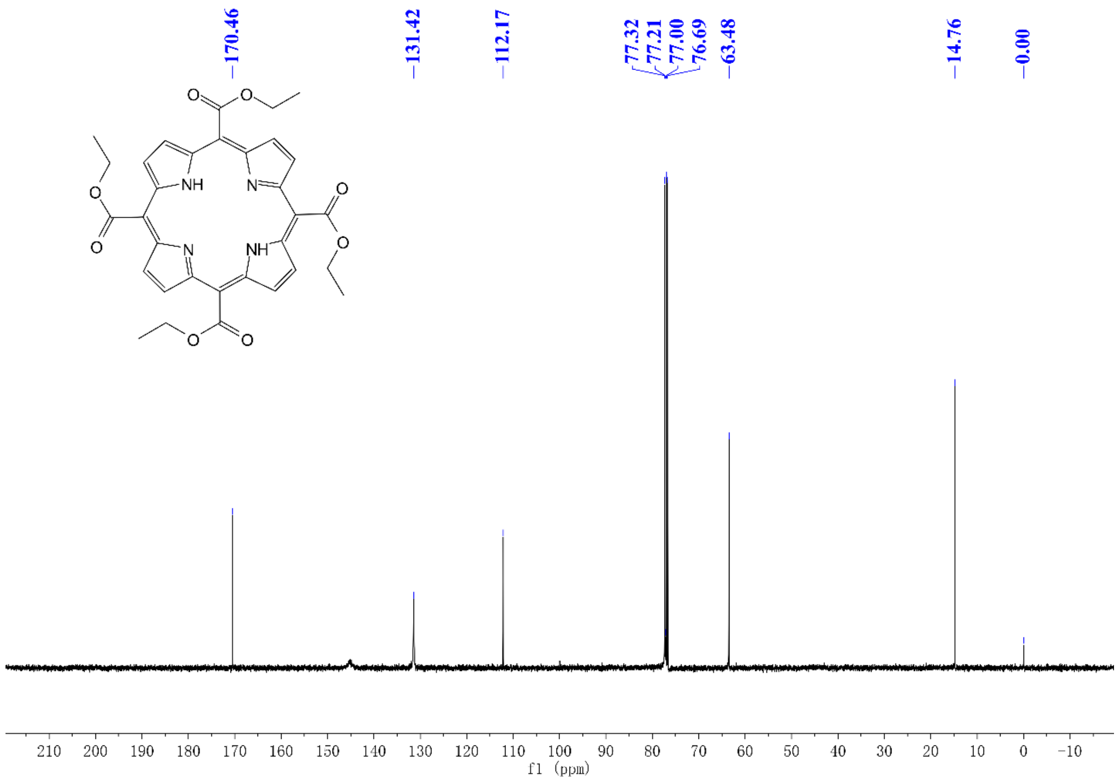

Figure S5. ^{13}C NMR of 5, 10, 15, 20-tetrakis(ethoxycarbonyl)porphyrin (**1**).

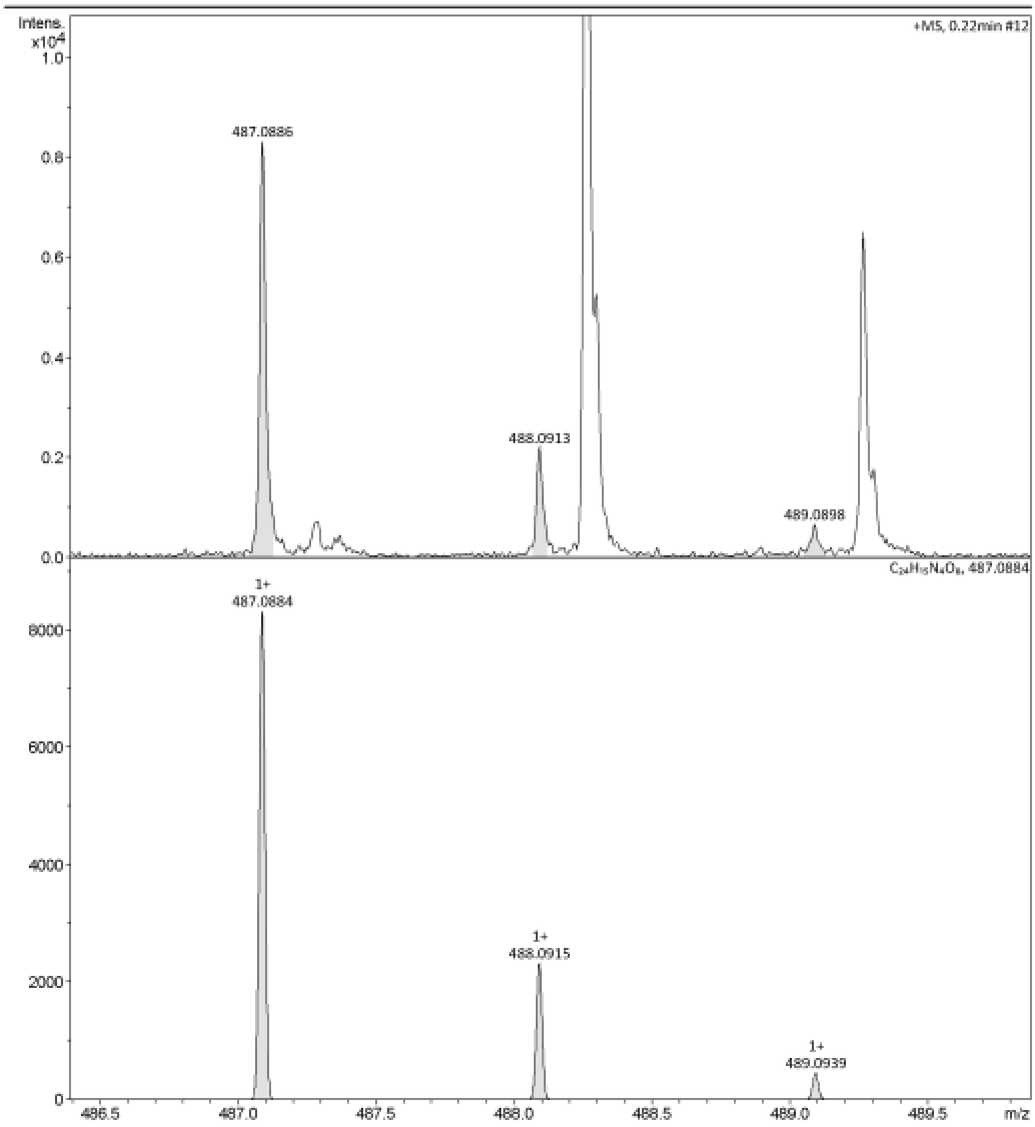

Figure S6. HR-MS of 5, 10, 15, 20- tetrakis (carboxyl) porphyrin (**2**)

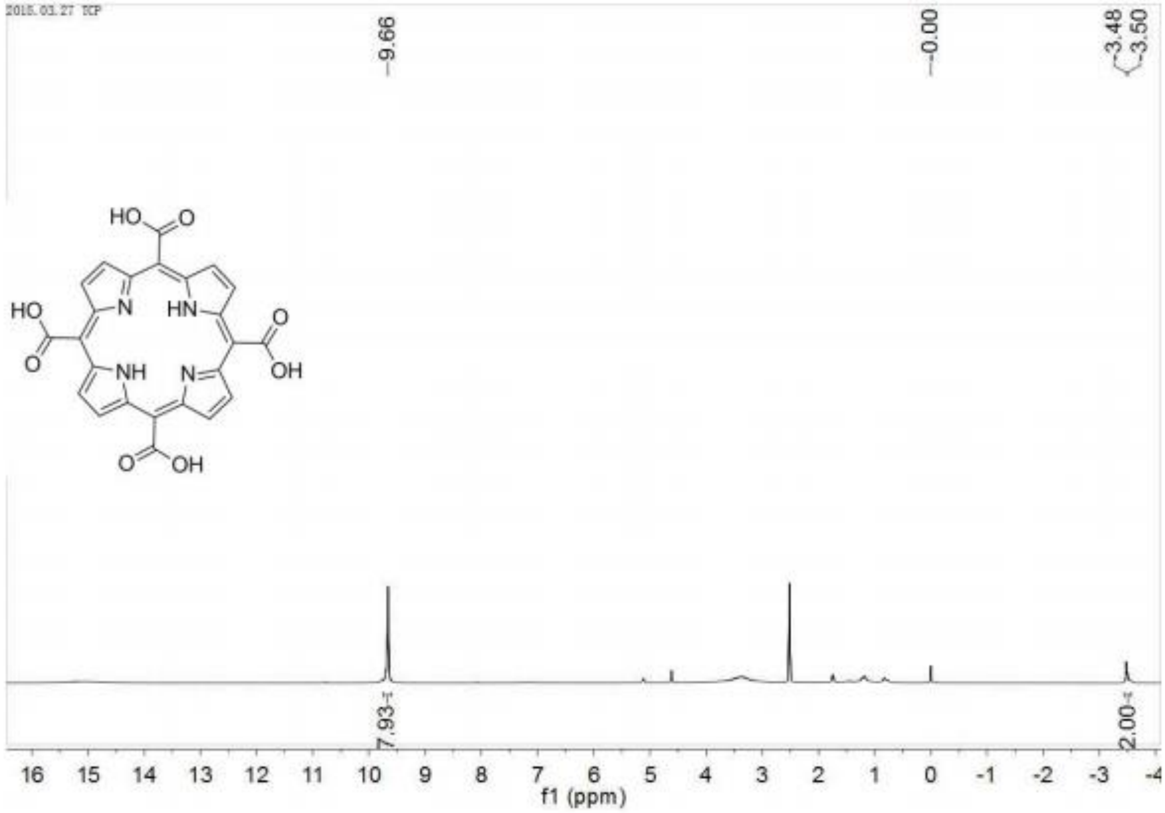

Figure S7. ^{1}H NMR of 5, 10, 15, 20- tetrakis (carboxyl) porphyrin (**2**)

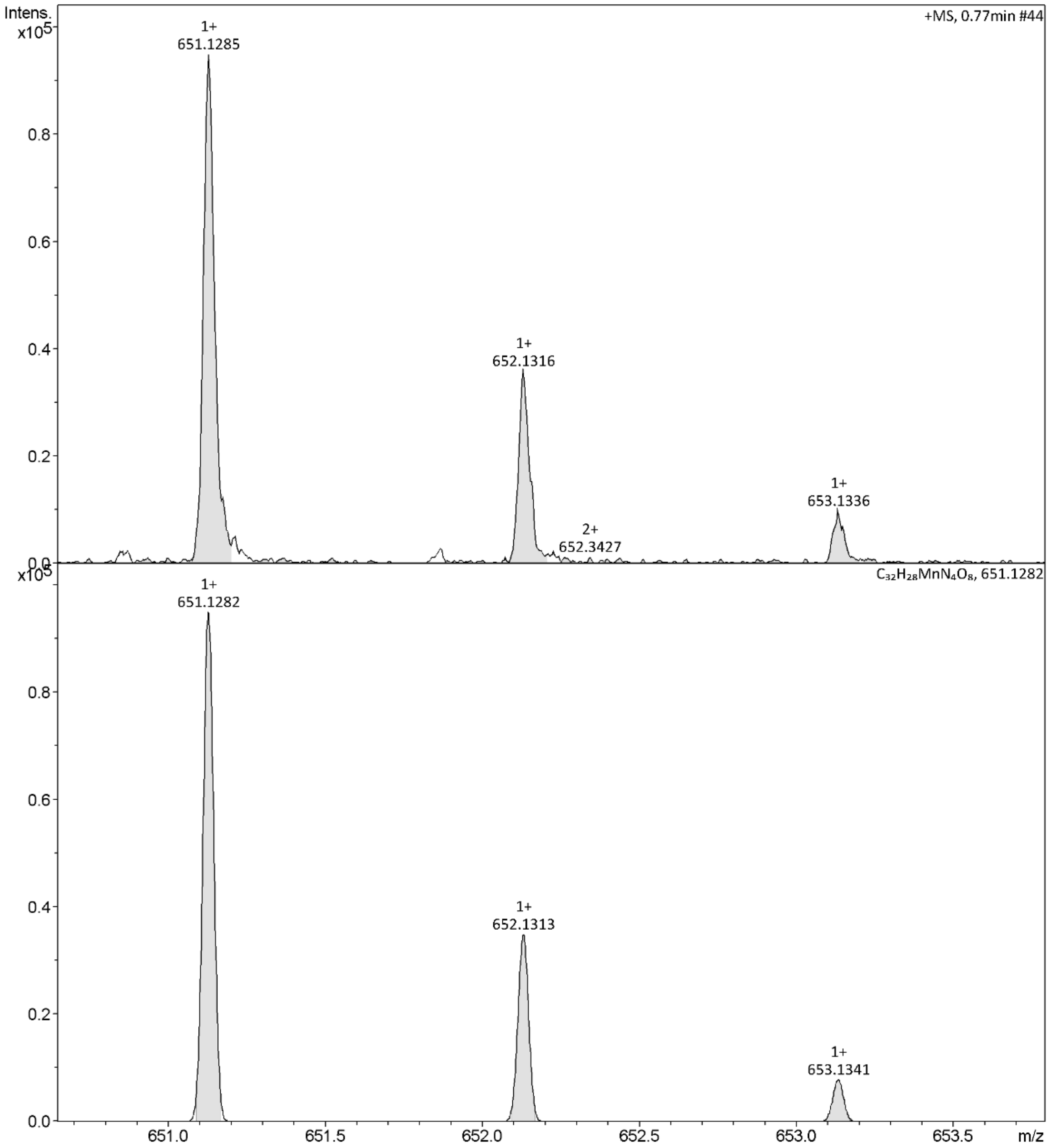

Figure S8. HR-MS of 5, 10, 15, 20-tetrakis(ethoxycarbonyl)porphyrin manganese (III) (**1-Mn**)

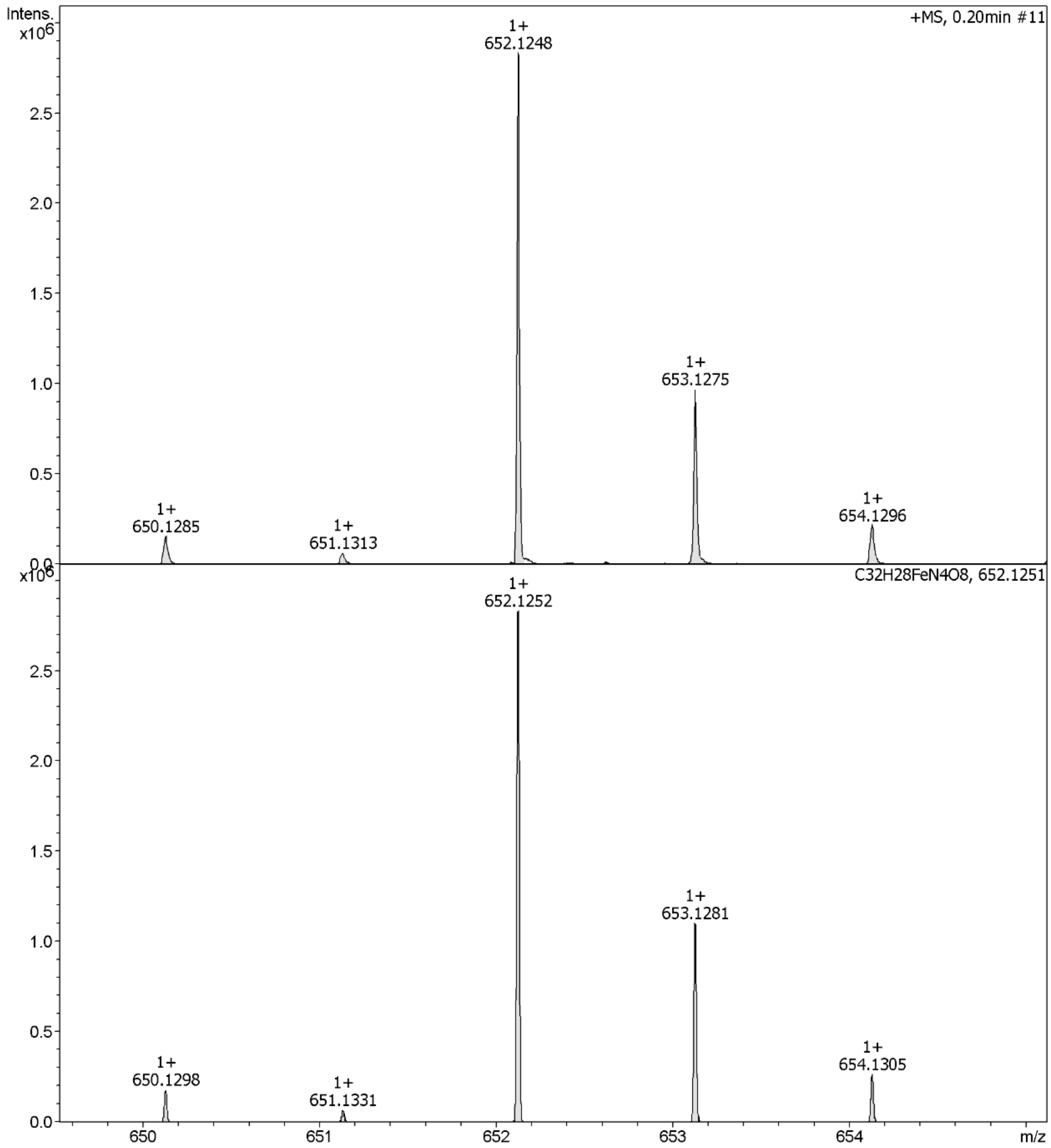

Figure S9. HR-MS of 5, 10, 15, 20-tetrakis(ethoxycarbonyl)porphyrin iron (III) (**1-Fe**)

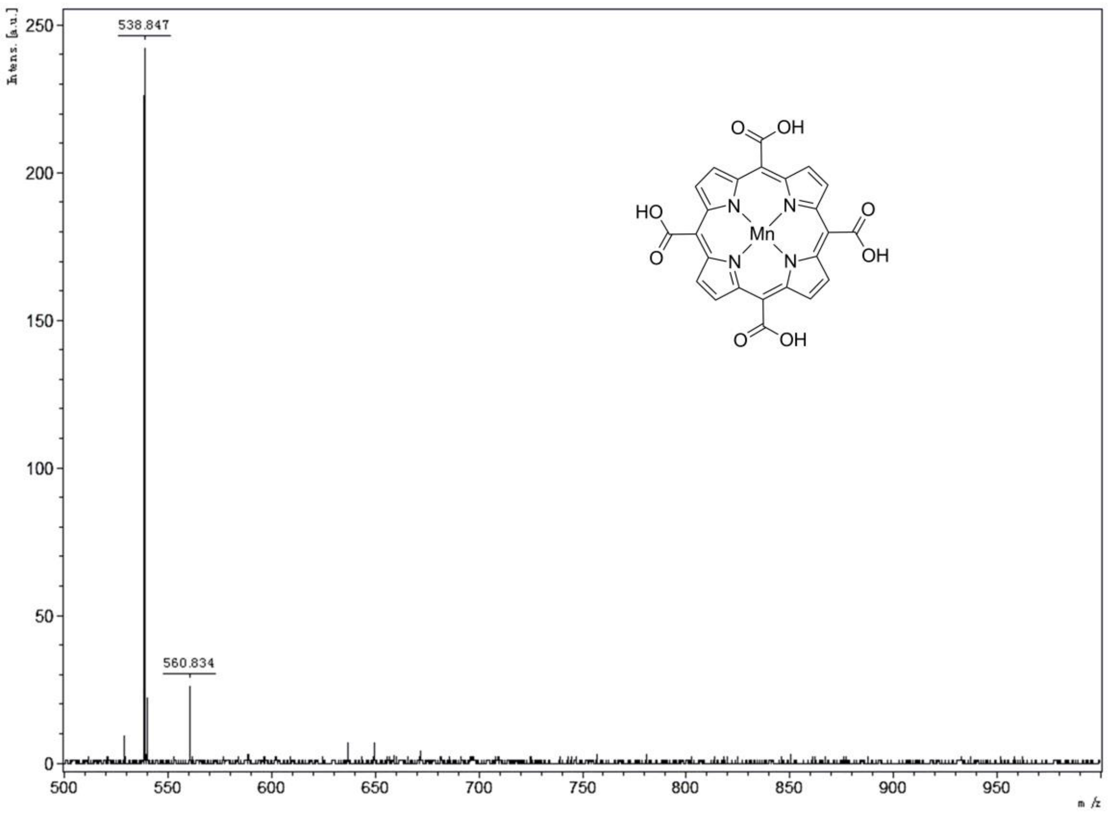

Figure S10. MALDI-TOF MS of 5, 10, 15, 20-tetrakis(carboxyl)porphyrin manganese (III) (**2-Mn**)

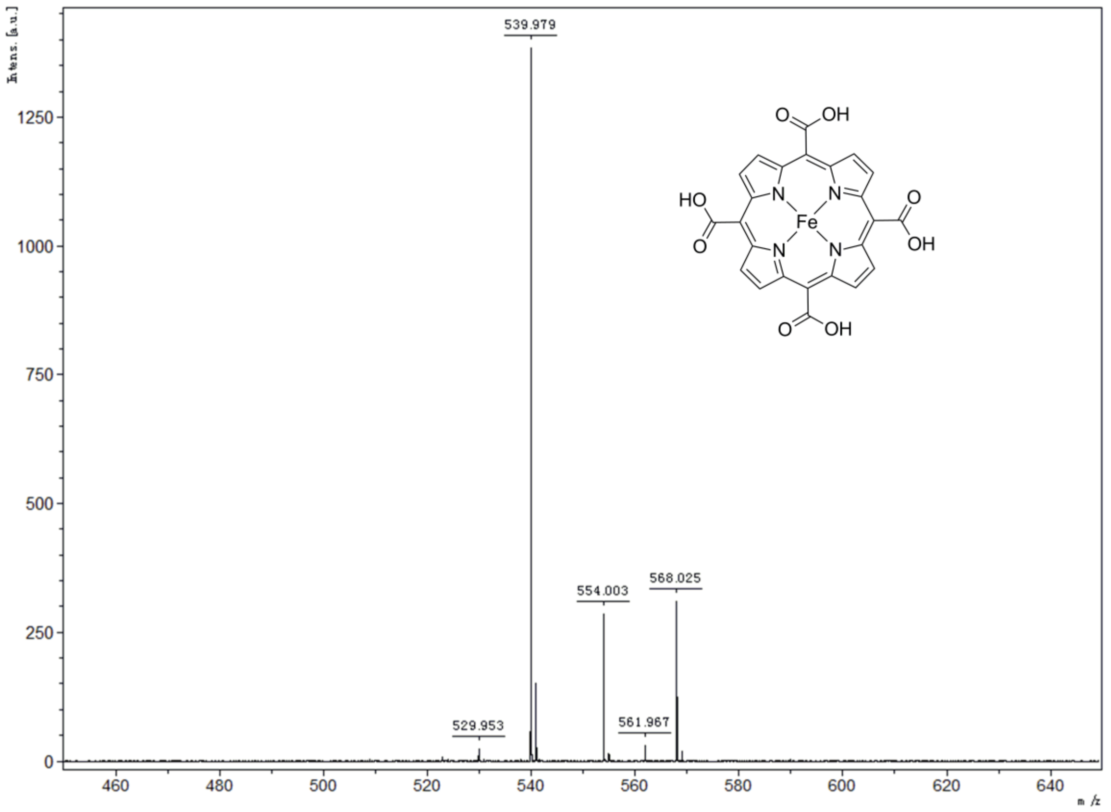

Figure S11. MALDI-TOF MS of 5, 10, 15, 20-tetrakis(carboxyl)porphyriniron (III) (**2-Fe**)

. **HPLC trace of porphyrins 2, 2-Mn and 2-Fe**

Reversed-phase HPLC analysis: The samples were analyzed on AT. ChromC[18] column (4.6×250mm).

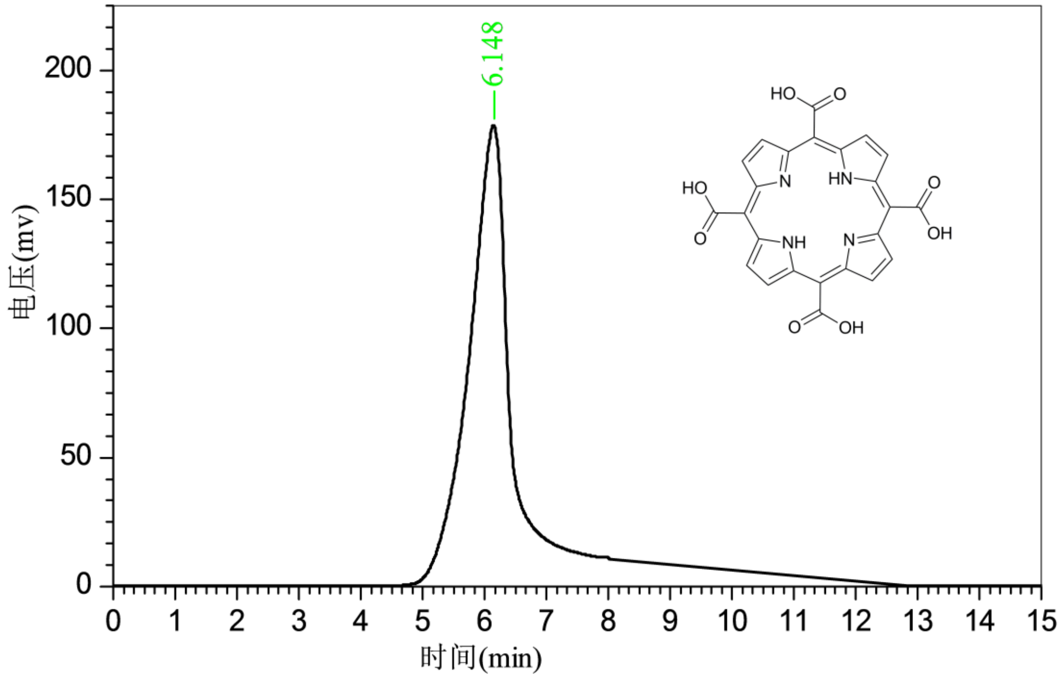

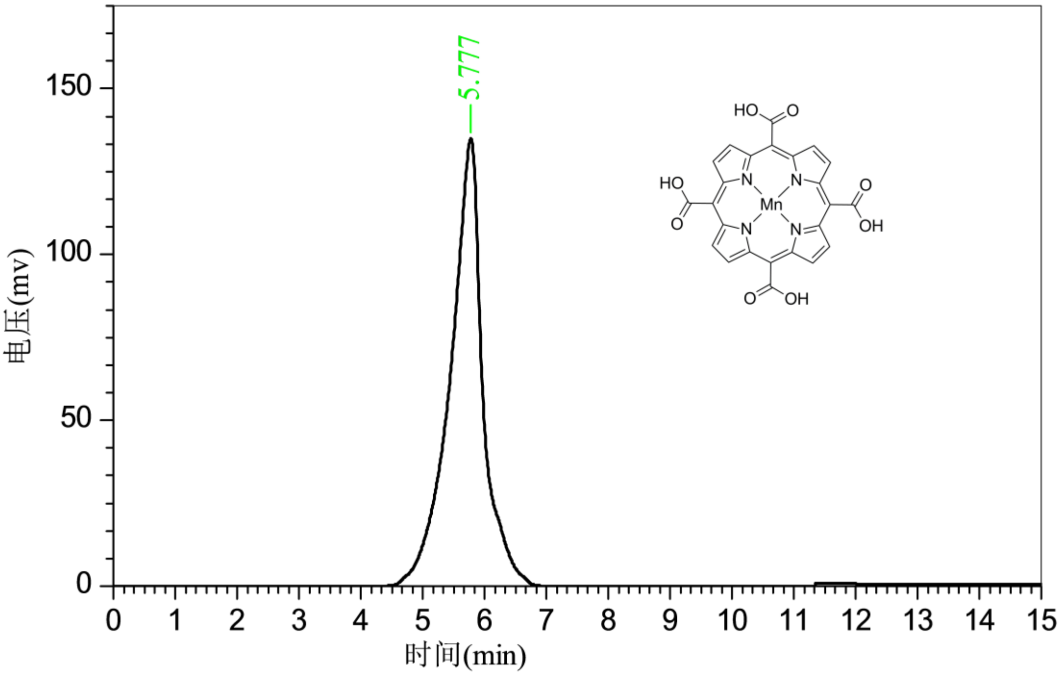

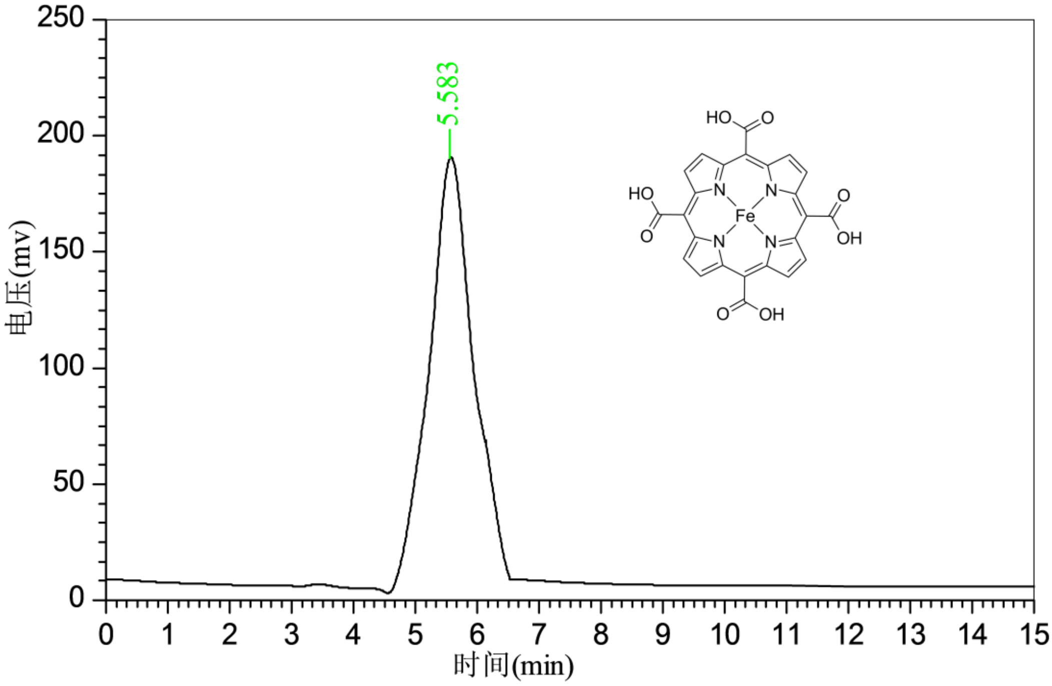

Figure S12. Porphyrin **2**, **2-Mn**, **2-Fe**, detection at 400 nm, CH_3OH : H_2O 95 : 5, 0.3 mL/min, retention time is 6.148, 5.777, 5.583min, respectively.

3. The UV-Vis spectra changes of 2-Mn (a) and 2-Fe (b) upon the addition H_2O_2

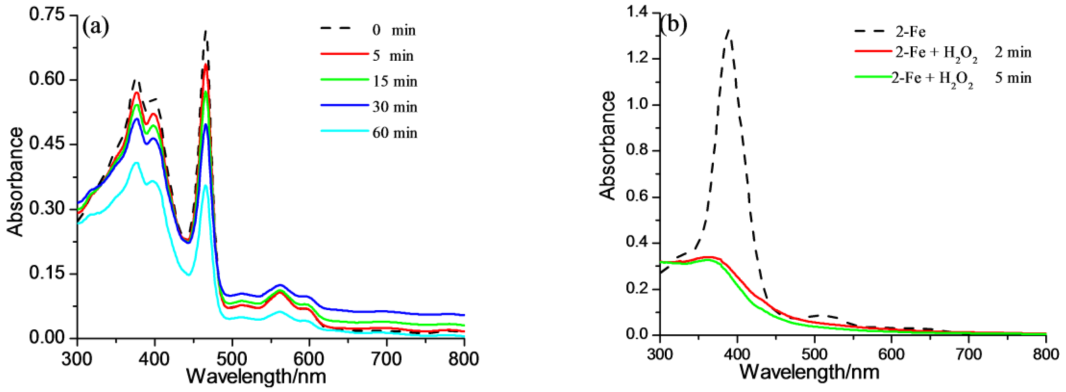

Figure S13. The UV-Vis spectra changes of **2-Mn** (**a**) and **2-Fe** (**b**) upon the addition H_2O_2 at different time. The arrow shows the absorbance along with the change of time. [**2-Mn**] = [**2-Fe**] = 30 μM, [H_2O_2] = 20 mM.
